# Access to healthcare for disabled individuals: An analysis of judgments of the European Court of Human Rights from an ethical perspective

**DOI:** 10.3389/fpubh.2022.1015401

**Published:** 2023-01-10

**Authors:** Tobias Skuban-Eiseler, Marcin Orzechowski, Florian Steger

**Affiliations:** ^1^Faculty of Medicine, Institute of the History, Philosophy and Ethics of Medicine, Ulm University, Ulm, Germany; ^2^kbo-Isar-Amper-Klinikum Region München, München-Haar, Germany

**Keywords:** disabled persons, access to healthcare, medical ethics, international law, human rights

## Abstract

**Introduction:**

Individuals with disabilities (ID) suffer from restricted access to healthcare. This contributes to their poorer health status and constitutes an ethical challenge. The aim of this research was to systematically analyze judgments of the European Court of Human Rights (ECtHR) to illustrate examples of restricted access to healthcare for ID.

**Methods:**

Through a search in the ECtHR's database we identified judgments dealing with access to healthcare for ID. The search resulted in *n* = 329 judgments, of which *n* = 55 were included in the analysis. A descriptive statistic was performed on Articles of the European Convention on Human Rights and violation of these articles. Qualitative thematic analysis was conducted to group the judgments in thematic categories.

**Results:**

Most applications were filed against Russia (*n* = 23), followed by Poland (*n* = 8) and Ukraine (*n* = 7). The youngest applicant was 18, the oldest 72 years old. An overwhelming majority of cases dealt with disabled prisoners. Most of the judgments involved Article 14 and Article 8. We identified seven partially overlapping categories representing thematic patterns in the analyzed judgments.

**Discussion:**

Any restriction of access to healthcare can be considered a violation of human rights. However, the results show a relatively low total number of judgments dealing with limited access to healthcare for ID. This could be a further confirmation of the fact that ID still experience too little attention in our societies. Especially in the context of detention, ID is restricted from receiving the healthcare they require. Indirect ways of a restricted access to healthcare should not be overseen.

## 1. Introduction

There are at least 1 billion individuals with disabilities (ID) worldwide, which corresponds to about 15% of the world's population (1). Originally, disability was seen as an illness that had to be diagnosed and treated and thus primarily fell within the sphere of the action of medicine. With the establishment of the social model of disability in the 1970s, disability has increasingly been considered a result of social limitations, making it society's task to remove these limitations (2). The Convention on the Rights of Persons with Disabilities (CRPD) of the United Nations (UN) conceives disability as resulting from an interaction between persons with limitations on the one hand and social limitations on the other hand. Therefore, the task to alleviate the restrictions of ID in numerous areas of life shall be assigned both to medicine and to society (3).

In the CRPD, the UN declared that ID have the right to receive the same range and quality of healthcare as other persons. Furthermore, the text of the CRPD explicitly states that there must be no restriction of access to healthcare for ID (3). Despite this, the preservation of their dignity and autonomy and the abolition of inequalities for ID have been demanded at least since the 1960s (4). The current COVID-19 pandemic once again illustrated that the interests of ID in accessing healthcare are not being considered to the same extent as those of other persons (5). To counteract the danger of discrimination against ID, the German Federal Constitutional Court even felt compelled in December 2021 to stipulate that ID must not be disadvantaged if a triage would be necessary (6).

Restricted access to healthcare of ID represents a serious social limitation (7, 8). Various factors contribute to a limited access to healthcare for ID: stigmatization (9), comorbidities and difficulty in communication (10), misconceptions and negative attitudes toward ID (11), lack of knowledge or attention on part of healthcare providers (11, 12), insufficient research activities (13), lack of integration of disability-specific content in medical curricula (14), difficulties regarding the transport of ID to medical facilities and associated high costs (7, 11), insufficient flexibility of the medical care system that frequently overlooks specific needs of ID (12), poverty (11), barriers in access to medical facilities (15), and a lack of integration of the voices of ID in service design (8).

As ID often have higher healthcare needs (16), limited access to healthcare certainly contributes to the poorer health status of ID than those without disabilities (1). From the ethical point of view, the right to health is one of the human rights, inseparably connected with the ethical principles of human dignity and social justice. This standpoint is represented and anchored in numerous international treaties, i.e. the Universal Declaration of Human Rights (17), and the Constitution of the World Health Organization (18).

The European Court of Human Rights (ECtHR) is the instance that deals with violations of human rights when the legal instances of the respective member state have been exhausted. Thus, the ECtHR's judgments are normative acts dealing with central medico-ethical questions of human dignity, justice, and equity in access to healthcare. The jurisdiction of the ECtHR should be binding for the 46 member states of the Council of Europe. The judgments of the ECtHR include not only legal but also normative considerations on central ethical issues. They can thus serve as a valuable source to explore in what ways access to healthcare for ID is restricted in individual member states. Therefore, the aim of this research was an analysis of the judgments of the ECtHR with consideration of the following questions: (i) How many ECtHR judgments deal with a restriction of access to healthcare for ID? (ii) How can these judgments be grouped thematically? (iii) How does the ECtHR assess these cases, in particular regarding their ethical content?

## 2. Materials and methods

We used HUDOC, a database of the ECtHR's case law, to retrieve all relevant judgments for our study (accessible under https://hudoc.echr.coe.int/eng#{%22documentcollectionid2%22:[%22GRANDCHAMBER%22,%22CHAMBER%22]}). On 4th January 2022, we performed a search with the following search terms' combinations: “disability” and “access” and “healthcare”, or “disability” and “access” and “health care”, or “disabled” and “access” and “healthcare”, or “disabled” and “access” and “health care”.

**Figure 1:**
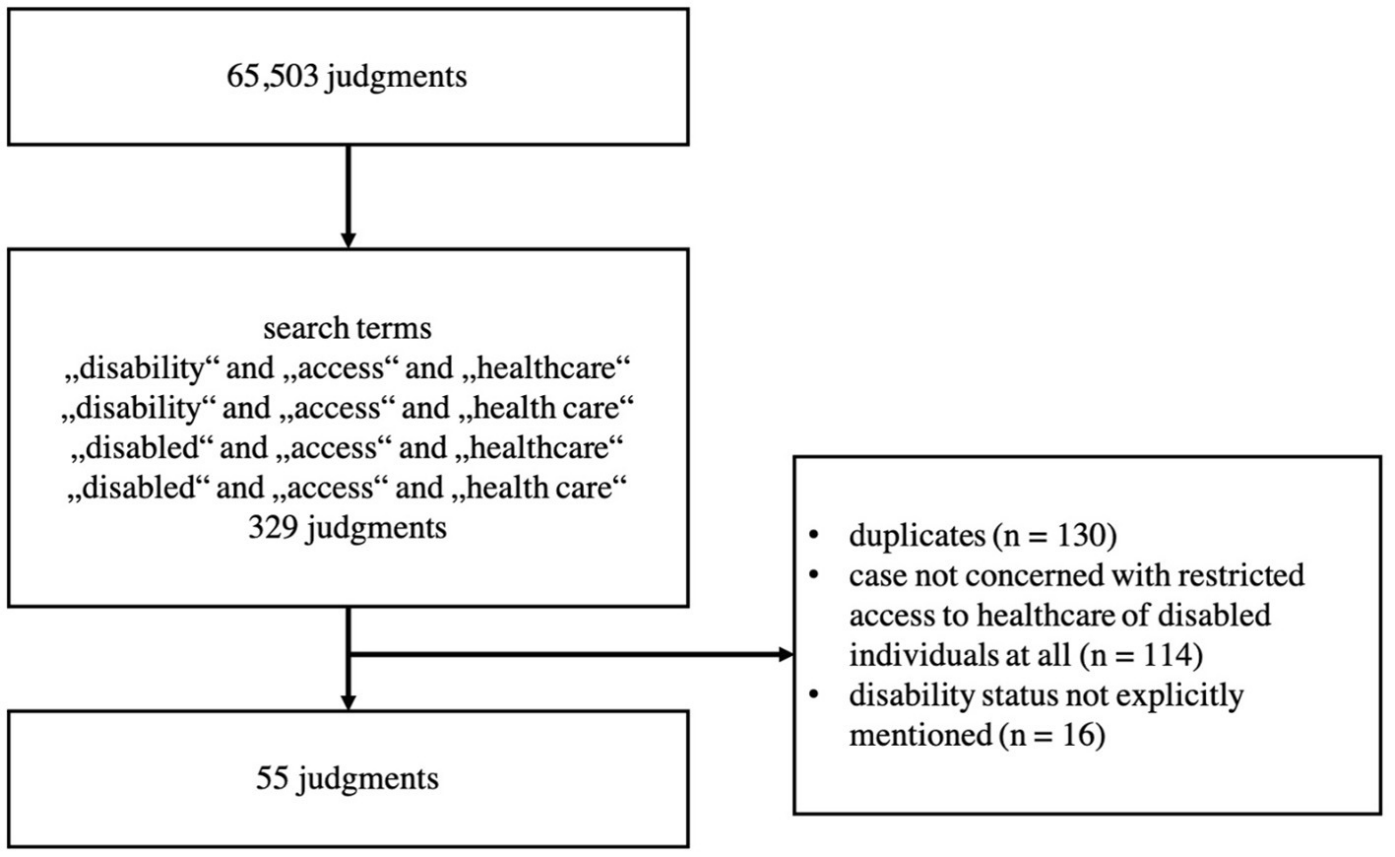
Flowchart of the search.

Our search yielded *n* = 329 judgments. *N* = 130 duplicates could be identified and eliminated. The remaining *n* = 199 judgments were read thoroughly to examine their relevancy with respect to our research questions. We identified *n* = 55 judgments relevant to our investigation. We excluded *n* = 144 judgments that were either not concerned with restricted access of ID to healthcare at all or in which the disability status of the applicant was not explicitly mentioned (Figure 1). We only included judgments that clearly dealt (1) with individuals that have been granted a disability status and (2) restricted access to healthcare of those individuals.

### 2.1. Descriptive statistics

We performed descriptive statistics on the Articles of the Convention that were involved in the judgments. All articles which the ECtHR ruled on as well as violation of these articles were counted. The judgments of the ECtHR often involve different articles in one case or even different paragraphs of the same article. For example, a judgment could state that one sub-paragraph of an article was violated while other sub-paragraphs were not breached or the substantive aspect of an article was violated while the procedural aspect was not. We counted a violation of an article if at least one violation of one of its sub-paragraphs or aspects was held by the ECtHR. This procedure involves a certain limitation of our investigation.

### 2.2. Thematic analysis

We performed a thematic analysis on all 55 relevant judgments. This is a quantitative approach for identification of recurring themes or patterns in narrative or text materials (19, 20). We inductively formulated and critically discussed thematic categories that could be derived from the analysis of the textual content of the judgments. Or research team was multiprofessional and included a psychiatrist (T.S.), a physician and expert in the history, philosophy and ethics of medicine (F.S.), and a political scientist (M.O.). The identified categories represent important thematic patterns of the judgments with regard to the research aim and research questions and do not depend exclusively on quantifiable measurements. We illustrate each of the categories by presenting representative examples of the analyzed judgments.

## 3. Results

### 3.1. Countries and time period of analyzed judgments

The *n* = 55 judgments derived from *n* = 15 countries. The most applications were filed against Russia (*n* = 23), followed by Poland (*n* = 8) and Ukraine (*n* = 7) (Table 1). Although the database of the ECtHR contains judgments from the year 1960 on, we could only identify judgments deriving from the time period between 2001 and 2021. With regard to the annual number of judgments during this period, we could not identify any trends or visible patterns.

**Table 1:** Overview of the countries the analyzed judgments derive from and numbers of analyzed judgments.

**Country**	**No. of judgments**
Russia	23 (41.82%)
Poland	8 (14.55%)
Ukraine	7 (12.73%)
United Kingdom	3 (5.45%)
Romania	3 (5.45%)
Switzerland	2 (3.64%)
Serbia	1 (1.82%)
Denmark	1 (1.82%)
Greece	1 (1.82%)
Bulgaria	1 (1.82%)
Czech Republic	1 (1.82%)
Germany	1 (1.82%)
Latvia	1 (1.82%)
Belgium	1 (1.82%)
Lithuania	1 (1.82%)

### 3.2. Age of applicants at the time of the alleged violation of the convention

**Figure 2:**
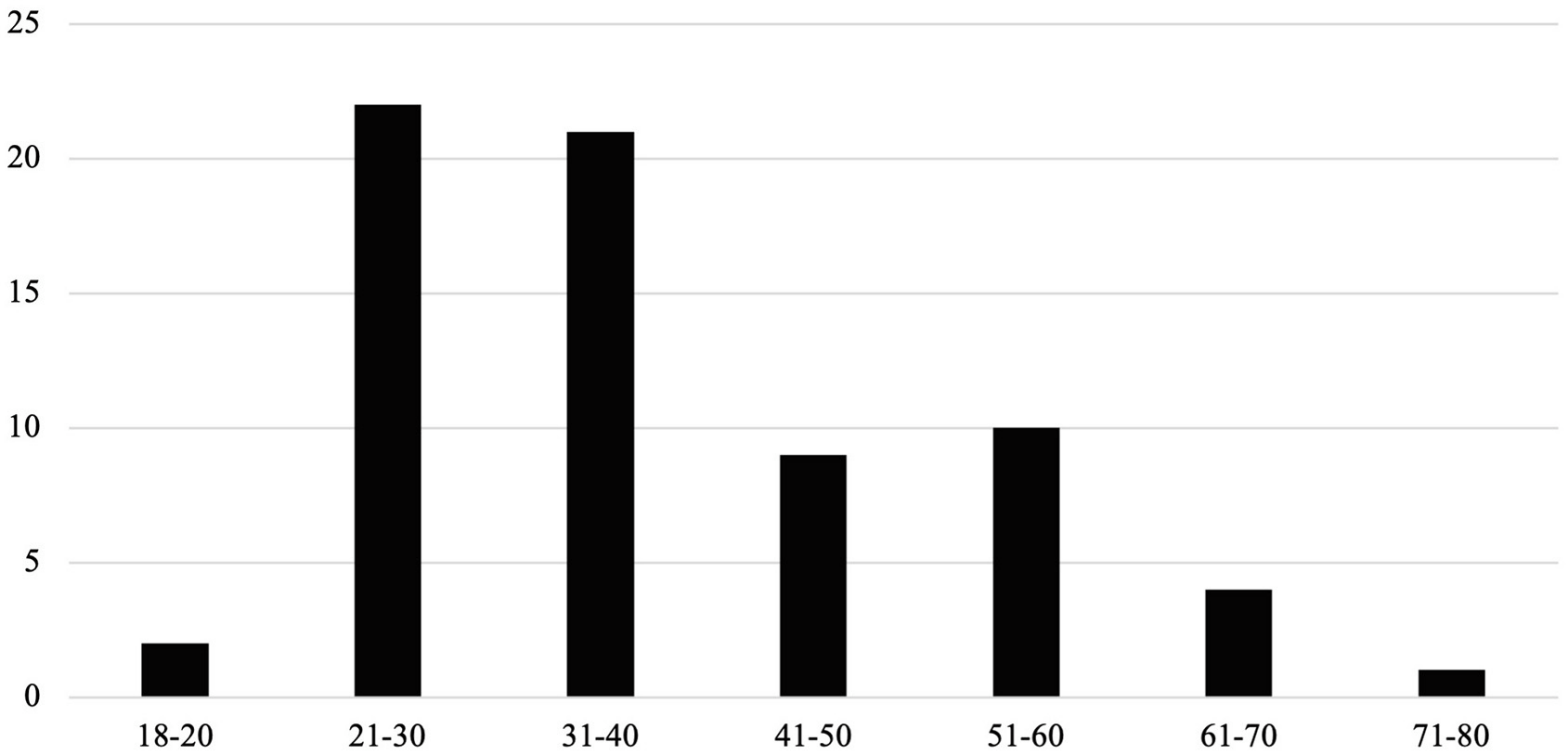
Distribution of age of applicants in our analyzed judgments (2001–2021). On the horizontal axis, we depict age frames, and on the vertical axis the number of judgments.

We analyzed all judgments regarding the age of the applicants at the time of the first alleged violation of an Article of the ECtHR. In the case of detained applicants appealing against the circumstances of the detention, we applied the date of the placement into detention; in all other cases, we used the point of time we first found a hint to an alleged violation of the Convention. As some judgments are dealing with more than one applicant, the sum of all applicants in our analyzed judgments is 70. In one case we were not able to determine the age of the applicant. The youngest applicant was 18, the oldest 72 years old. We did not find any cases dealing with applicants younger than 18 years (Figure 2).

### 3.3. Attribution of applicants to other minority groups

In *n* = 43 cases (78.18%) we could attribute the applicants not only to the minority group of ID but also to other minority groups. In *n* = 2 judgments (3.64%) the disabled applicant was a migrant respectively an inhabitant of a social care home. The overwhelming majority of judgments (*n* = 41, 74.55%) dealt with applicants that were disabled prisoners.

### 3.4. Articles of the European Convention on Human Rights

In Table 2 we present frequencies of the Articles of the Convention involved in the analyzed judgments. Most of the judgments involved Article 14 (prohibition of discrimination) and Article 8 (right to respect for private and family life).

**Table 2:** Frequencies of articles on rights and freedom (Section 1, Articles 1–18) and protocols of the European Convention on Human Rights in the *n* = 55 judgments included into this analysis.

**Articles of the European Convention on Human Rights**	**Name of the article**	**Judgments involving this article**	**Judgments in which at least one violation of this article was found (either alone or in conjunction with other articles)**
Article 1	Obligation to respect human rights	*n* = 1 (1.82%)	*n* = 0
Article 2	Right to life	*n* = 1 (1.82%)	*n* = 1 (100%)
Article 3	Prohibition of torture	*n* = 47 (85.45%)	*n* = 38 (80.85%)
Article 5	Right to liberty and security	*n* = 16 (29.09%)	*n* = 8 (50%)
Article 6	Right to a fair trial	*n* = 18 (32.73%)	*n* = 9 (50%)
Article 8	Right to respect for private and family life	*n* = 17 (30.91%)	*n* = 7 (41.18%)
Article 13	Right to an effective remedy	*n* = 16 (29.09%)	*n* = 11 (68.75%)
Article 14	Prohibition of discrimination	*n* = 7 (12,73%)	*n* = 1 (14.29%)
Article 17	Prohibition of abuse of rights	*n* = 2 (3.64%)	*n* = 0
Article 34	Individual applications	*n* = 5 (9.09%)	*n* = 4 (80%)
Art 1 of Protocol No. 1	Protection of property	*n* = 4 (7.27%)	*n* = 1 (25%)
Art 1 of Protocol No. 12	General prohibition of discrimination	*n* = 1 (1.82%)	*n* = 0
Art 2 of Protocol No. 7	Right of appeal in criminal matters	*n* = 1 (1.82%)	*n* = 0

### 3.5. Categories

**Figure 3:**
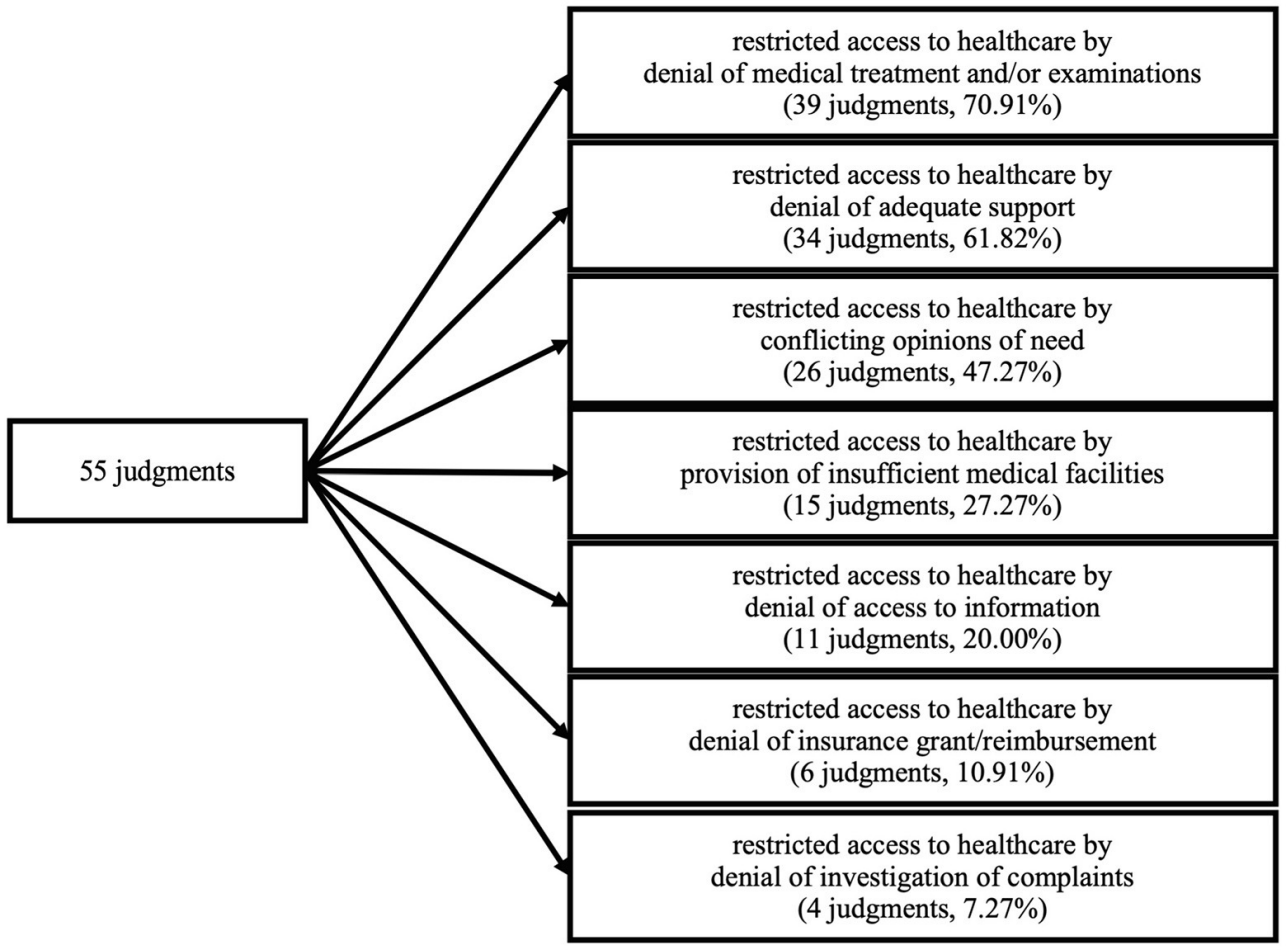
General count and percentages of judgments we found regarding each category.

We identified seven partially overlapping categories representing thematic patterns in the analyzed judgments (Figure 3). These categories describe in what way access to healthcare is restricted for ID. The categories are not mutually exclusive – often judgments were classified as belonging to several categories. The access to healthcare for ID was found to be restricted by (i) denial of medical treatment and/or examinations (*n* = 39, 70.91%), (ii) denial of adequate support (*n* = 34, 61.82%), (iii) conflicting opinions of need (*n* = 26, 47.27%), (iv) provision of insufficient medical facilities (*n* = 15, 27.27%), (v) denial of access to information (*n* = 11, 20%), (vi) denial of insurance grant/reimbursement (*n* = 6, 10.91%) and (vii) denial of investigation of complaints (*n* = 4, 7.27%). We regard all these categories as phenomena that are either directly restricting the access to healthcare for ID (categories i, ii, iii, vii) or that exclude ID from full participation in the medical healthcare system (categories iv, v, vi).

#### 3.5.1. Judgments involving a restriction of access to healthcare for ID by denial of medical treatment and/or examinations

As an example might serve case *Reshetnyak v. Russia* (appl. no. 56027/10). This case deals with an applicant that is arrested for robbery. Suffering from tuberculosis, he was admitted to a correctional colony primarily occupied by inmates infected with tuberculosis. Although he was examined by prison doctors on several occasions, his state of health deteriorated constantly. Even though he proved to be smear-positive (meaning that the bacterium causing tuberculosis could be detected in his sputum), he was not provided with drugs that had a serious prospect of improving his health status. Instead, the prison doctors kept administering the same drugs in various combinations that had proved to be ineffective (amongst others, antihistamines, multivitamins and muscle relaxants, which have no effect on tuberculosis). Moreover, there were long delays between deteriorations of the applicant's health and the reaction of the colony doctors. Finally, the applicant was diagnosed with a destructive tuberculosis and a tubercular intoxication. Only after about 5 years, a much-needed drug susceptibility testing was performed by the prison doctors, revealing the applicant to suffer from multi-drug-resistant tuberculosis, tempting them to acknowledge that the drug regimen administered so far was ineffective. In the opinion of the ECtHR, it is highly unsatisfactorily that the detention facility, which primarily was accommodated by tuberculosis patients, obviously lacked proper drugs to provide an adequate therapy. It stated that the mere fact that a prisoner is seen by a doctor and prescribed a treatment is not enough to qualify a detention facility's medical care as adequate. Moreover, the Court did not accept any problems concerning the supplementation with effective drugs on the side of the detention facility as excuse for not being able to provide the healthcare needed by the applicant. Hence, it saw a violation of Article 3 (besides Article 13).

#### 3.5.2. Judgments involving a restriction of healthcare for ID by denial of adequate support

We regard “support” as having a broader meaning than “medical treatment”: While “support” incorporates any form of specific attention a disabled individual may need, “medical treatment” refers to specific interventions usually ordered by a medical doctor.

Example: The applicant in the case *Patranin v. Russia* (appl. no. 12983/14) suffered from progressive multiple sclerosis and was recognized as being disabled. In 2012, he was arrested on suspicion of several crimes, including murder. He then experienced a significant deterioration of his health. Finally, he was found to suffer from left-sided hemiplegia, right-sided hemiparesis, partial atrophy of the visual nerves, symptomatic epilepsy, arterial hypertension and myopathy of both eyes. Although the prison doctors stated he should be released early on health grounds, his respective request was dismissed. He mentioned that he relied on constant assistance which he was in no way provided within the penal system. Being severely restricted in his movement, he had to stay in bed all day long. This was due to the fact that he even needed help when he wanted to be placed in his wheelchair. He had to endure significant unsanitary conditions as he had not been bathed for months, although he suffered from involuntary urination due to urethral dysfunction. Moreover, he received food only once a day as he was not able to eat or drink unaided. Being not able to defecate and being provided with an enema only every fortnight he had to bear severe pain. In numerable occasions, he did not receive necessary medical treatments. He was only released in 2015 after extensive proceedings concerning his state of health which was not compatible with the care the penal system provided. In its judgement, the ECtHR stated that any torture or inhuman or degrading treatment is prohibited regardless of the applicant's behavior and the circumstances. Such treatment would violate a person's human dignity. Moreover, also in prison anything should be done to treat health issues of a detainee or to prevent their aggravation. In the case at hand, the applicant was exposed to such inhuman and degrading treatment diminishing his human dignity. Therefore, the ECtHR stated a violation of Article 3 (in addition to a breach of articles 13 and 34).

#### 3.5.3. Judgments involving a restriction of access to healthcare for ID by conflicting opinions of need

In our analysis, we considered conflicting opinions of need as a situation, in which the applicant claimed specific care that others did not provide, as they seemed to have a different interpretation with respect to the care required. Basically, any denial of medical treatment or support can be reduced to conflicting opinions regarding the specific need of a person. However, we use this category separately and in contrast to the categories (i) “denial of medical treatment” and (ii) “denial of adequate support” to emphasize that in some cases the restriction of access to healthcare is the result of different and conflicting assessments regarding the specific need of an ID.

Example: In the case *G. v. Russia* (appl. no. 42526/07) the applicant was suffering from severe rectal cancer (amongst other illnesses) and was arrested on suspicion of committing bank fraud. Although his state of health deteriorated significantly during his time in prison, he was not released on medical grounds. About a month after his arrest, a large part of his sigmoid colon prolapsed which caused not only fecal incontinence, but also severe pain. He even lost consciousness on several times during his detention due to the intensity of pain he had to endure. He was not provided with absorbent briefs and was hardly able to perform basic hygiene measures he urgently needed. Although his situation worsened constantly and independent medical specialists demanded at several times that the applicant needed colorectal surgery, the prison doctor found his condition satisfactory not calling for immediate surgery. In the end, surgery was delayed for nearly a year. The ECtHR stated in its judgment that the medical problems of the applicant were not sufficiently addressed which not only led to a significant deterioration of the applicant's quality of life, but also had to be regarded as life-threatening. Moreover, the authorities did not provide adequate alternative treatment to ameliorate the applicant's suffering. Furthermore, denying the applicant the much-needed absorbent briefs caused significant distress and embarrassment for the applicant. Consequently, the Court saw a breach of Article 3 (in addition to a violation of Article 5).

#### 3.5.4. Judgments involving a restriction of access to healthcare for ID by provision of insufficient medical facilities

Insufficient medical facilities could imply the lack of necessary specialists (as in *Bubnov v. Russia*, appl. no. 76317/11), the impossibility to provide required medication (as in *Petukhov v. Ukraine*, appl. no. 41216/13), the lack of medical equipment (as in *G. v. Russia*, appl. no. 42526/07), being kept together with infectious patients (as in *Romokhov v. Russia*, appl. no. 4532/04) and even syringes used more than once (*Ukhan v. Ukraine*, appl. no. 30628/02).

Example: The applicant in *Gurenko v. Russia* (appl. no. 41828/10) suffered two myocardial infarctions before being arrested for beating his female partner to death. Although his medical condition already was unsatisfactory before the arrest and even deteriorating while being in detention, he never was examined by a cardiologist. The medical professions in charge of him included general physicians, a dermatologist, a surgeon, an otolaryngologist, a tuberculosis specialist, a drug addiction specialist, an ophthalmologist and a psychiatrist. Due to lack of knowledge in cardiology, they only were able to treat his problems symptomatically. A significant number of medicines necessary to treat the applicant's medical problems, were not provided by the detention facility, but had to be bought and brought to the applicant by his son. On several occasions, required tests (like ultrasound scanning) were not provided or could not be interpreted (ECG testing). Furthermore, essential recommendations by the attending physician of the prison hospital the applicant was admitted to during his detention, were not followed. Even the prison hospital was not able to provide emergency resuscitation assistance if need be. In its judgment, the ECtHR acknowledged that the applicant did not receive the required treatment and therefore experienced a degradation of his human dignity by persistent mental and physical suffering. Therefore, the ECtHR stated a violation of Article 3.

#### 3.5.5. Judgments involving a restriction of access to healthcare for ID by denial of access to information

We regard a denial of access to information as a form of restricted access to healthcare since full and autonomous participation in any healthcare-related aspect for any patient is dependent on being informed about all necessary circumstances as much as possible. Without being informed about the nature of the medical condition at hand and about interventions that might be possible or that even have taken place, a patient cannot perform an informed consent and cannot adequately claim his or her rights (e. g. during legal procedures). Hence, full access to all relevant information and all medical files is an indispensable aspect of a patient's access to healthcare.

We found several ways in which access to information could be denied: denial of access to information partly or at all (e. g. *Makshakov v. Russia*, appl. no. 52526/07), denial of assistance to read medical files although severe reduction of eyesight (*Tysiac v. Poland*, appl. no. 5410/03), illegible records or denial to record complaints (e. g. *Ukhan v. Ukraine*, appl. no. 30628/02) or discrepant files (e. g. *Reshetnyak v. Russia*, appl. no. 56027/10).

Example: The applicant of *I. N. v. Ukraine* (appl. no. 28472/08) was involuntarily placed in a psychiatric facility by the authorities. The respective documents that argued for the applicant's admission in a psychiatric facility had not been submitted to the Court. He was not allowed to study his medical files to learn about the legal basis of his being subjected to psychiatric treatment. Subsequently, he instituted proceedings against the psychiatric institutions he was placed in. The following proceedings were extensive and lengthy and lasted from 2001 to 2007. During that time, numerous court hearings were scheduled and, in many cases, postponed (due to failure of the defendants to appear in court, non-availability of the court recording equipment or expire of the term of office of the judge in charge of the applicant's case). Obviously, although the applicant could not receive all relevant information about his medical case, his employers had been informed about his state of health. Moreover, he was not informed about the type of medication he was given. In its judgement, the ECtHR argues that there would have been insufficient legal requirements regarding the placement of the applicant in a psychiatric facility. It criticized that it had not been provided with any evidence that the applicant was suffering from a mental disorder at the material time that would present a danger to him or to others justifying his hospitalization. The Court stated that the excessive length of proceedings was unreasonable and caused by facts the authorities were primarily responsible for. In its decision, the ECtHR saw a violation of Articles 5 and 6.

#### 3.5.6. Judgments involving a restriction of access to healthcare for ID by denial of insurance grant/reimbursement

As an example we provide case *Shmalko v. Ukraine* (appl. no. 60750/00). The applicant in this case is a disabled veteran of the Second World War suffering from myasthenia. He instituted proceedings seeking reimbursement of his costs for a drug he needed to treat his medical condition. Being not available in the Ukraine, he had to buy it abroad and cover the costs himself. After several rejections of his claim as being unsubstantiated, the Regional Court of Appeal allowed the applicant's claims in part. However, it lasted more than 1 year for the respective institutions to provide the applicant with the money the litigation demanded. In its argumentation, the ECtHR states that a State's alleged lack of funds cannot be an excuse for not executing a judgment. The State of Ukraine has the obligation to provide medication free of charge for the applicant. Moreover, the age of the applicant and his disability called urgently for an undue payment of his costs caused by his obtaining medication necessary to treat his medical condition. By causing a significant delay, the authorities furthermore prevented the applicant from a possession of his property. In conclusion, the ECtHR saw a violation of Article 6 and Article 1 of Protocol No. 1.

#### 3.5.7. Judgments involving a restriction of access to healthcare for ID by denial of investigation of complaints

We also regard a denial of investigation of complaints as a form of a restriction of access to healthcare. Full access to healthcare should incorporate a patient's possibility to claim for his or her rights regarding access to healthcare. Without this legal security, a patient would be restricted to passively waiting for access to healthcare to be provided and be restricted of the possibility to actively demand access to healthcare. Thus, the possibility to demand a proper investigation of complaints is a relevant part of full access to healthcare.

A denial of investigation of complaints was done in different ways: refusal of authorities to institute a proceeding to investigate an applicant's complaints (*Ukhan v. Ukraine*, appl. no. 30628/02), letters to Court not dispatched by authorities (*Sergey Babushkin v. Russia*, appl. no. 5993/08), denial of timely provision of information or complaints being non-answered at all (*Gurenko v. Russia*, appl. no. 41828).

Example: Case *Ukhan v. Ukraine* (appl. no. 30628/02) deals with an applicant who claimed to have suffered bodily injuries inflicted by police officers (fractured rib and major head injury) as he was forced to confess his offenses. Subsequently, the prosecutor's office refused to institute criminal proceedings to investigate the applicant's complaints referring on testimonies of the involved police officers. Also, the Regional Court of Appeal dismissed the applicant's appeal concerning ill-treatment. The applicant started suffering from severe headaches, lost mobility in his left side and a partial loss of sight in his left eye. A neurologist diagnosed him with a partial atrophy of the left eye nerve considered to be of traumatic origin. Although complaining about his medical problems and the insufficient medical care on numerous times (even by going on hunger strike), there was no adequate reaction of the authorities. They kept responding that his medical condition was satisfactory. The medical personnel even refused to examine and record his complaints. The ECtHR stated in its judgement that the adequacy of medical assistance provided to a detainee is, amongst others, dependent on the keeping of a comprehensive record including the detainee's medical status and the provided treatment. The Court criticized, that in the case of the applicant, significant aspects of his medical condition remained unreported and unsupervised and that the information provided in the medical records is incomplete. The Court decided that there was a breach of Article 3 and 13.

## 4. Discussion

### 4.1. Countries and time period of analyzed judgments

By far the highest number of our analyzed judgments challenged Russia (followed by a considerable margin by Poland and Ukraine). This is in line with the overall statistics of all judgments of the ECtHR: Russia is on second place (behind Turkey) in terms of the total number of judgments considered by the ECtHR, Ukraine is on fourth and Poland on sixth place (21). Numerous human rights violations have been known to take place in Russia (22). Although Russia signed the European Convention of Human Rights, the country often shows a profound disregard for the rulings of the European Court of Human Rights (23). Obviously, respect for the rights of ID is no exception. The fact that ID in Russia find ways to fight for their rights, gives some hope (24). Overall, the results show a relatively low total number of judgments dealing with limited access to healthcare for ID. This and the fact that the oldest judgments relevant to our research are just 11 years old could be a further confirmation of the fact that ID still experience too little inclusion and attention in our societies (25).

### 4.2. Age of applicants at the time of the (alleged) violation of an article of the ECtHR

The applicants in the analyzed judgments were all adults, the youngest applicant being 18 years old. An unexpected result is that we did not find any cases dealing with disabled children, considering that about one in ten children worldwide has a disability (26). This may reflect the fact that children with disabilities are one of the most neglected and marginalized groups frequently facing challenges in realizing their human rights (26). Regarding healthcare, this may be illustrated by the fact that cuts in services across Europe increased waiting times and lessened the time allotted for each child in the provision of healthcare (27). Even though we did not find a case dealing with disabled children, this does not in any way mean that there are no restrictions on access to healthcare for this group of people. It is rather the contrary: one could take the fact that no such cases end up at the ECtHR as an indication that there are (too) few legal processes regarding the access to healthcare of disabled children in the member states. Furthermore, only a small number of identified judgments were dealing with applicants older than 60. This could imply that older age might serve as additional factor of discrimination.

### 4.3. Attribution of applicants to other minority groups

An overwhelming majority of cases (*n* = 41, 74.55%) dealt with disabled prisoners. *N* = 22 (53.66%) of these cases concerned Russia. Various human rights violations in Russian prisons, particularly in relation to overcrowding and poor medical care, have already come to the attention of the ECtHR in numerous cases (28, 29). It is not surprising that ID are among these. However, it is remarkable that in most of these judgments the disability status did not play a central role in the argumentation of the ECtHR. Since the cases that come before the ECtHR are only the tip of the iceberg of all legal cases in a member state, this could be taken as a sign of a significant restriction of the legal rights of individuals who are “only” disabled.

### 4.4. Articles of the European Convention on Human Rights

Most judgments dealt with Article 3 (prohibition of torture, *n* = 47, 85.45%), followed by Article 6 (right to a fair trial, *n* = 18, 32.73%) and Article 8 (right to respect for private and family life, *n* = 17, 30.91%). Article 3, according to the ECtHR, expresses one of the most fundamental values of democratic societies. It prohibits ill-treatment regardless of the victim's conduct or the circumstances. The ECtHR considers not only “inhumane” conduct such as physical injury or the infliction of intense physical or mental suffering as criteria for ill-treatment, but also any “degrading” conduct that is likely to violate the human dignity of an individual or provoke feelings of fear, anguish or inferiority that may break a person's morals (*G. v. Russia*, appl. no. 42526/07 and *Stanev v. Bulgaria*, appl. no. 36760/06). Thus, any conduct that may violate the dignity of ID may in principle also constitute a violation of Article 3. In particular, the provision of adequate healthcare is seen as a positive obligation arising from Article 3 (29). However, to meet this obligation, adequate healthcare must not only be provided but must also be fully accessible (*Gurenko v. Russia*, appl. no. 41828/10). Thus, any restriction of access to healthcare can also be considered a breach of Article 3 and thus a violation of human rights. The numerous cases of restrictions on access to healthcare for ID depict violations of human rights and urgently need to be stopped. It should be borne in mind that restrictions on access to healthcare are not in every case obvious but often sublime and unnoticed.

### 4.5. Categories

In most of the analyzed judgments, access to healthcare was restricted in a very direct way, either by a denial of medical treatment and/or examinations (*n* = 39, 61.82%) or by a denial of adequate support (*n* = 34, 61.82%). The availability of only insufficient medical care (*n* = 15, 27.27%) and the restriction of access to healthcare due to conflicting opinions of need (*n* = 26, 47.27%) can also be considered direct restrictions. However, we also found less obvious ways in which ID are denied full participation in healthcare, such as a denial of access to information (*n* = 11, 20.00%), a denial of insurance grant/reimbursement (*n* = 6, 10.91%), or denial of investigation of complaints (*n* = 4, 7.27%). Healthcare should be considered as a concept that not only refers to a direct provision of medical interventions but also includes participation in ethical and legal aspects of the health system. This undoubtedly includes the right of patients to be fully informed, to be insured, and to take legal action if violations of their rights occur. Even though not all forms of disability are per se associated with an increased need for medical support, the risk for ID to suffer from health disorders is nevertheless very high: through secondary and co-morbid diseases, a greater vulnerability to age-related conditions, a higher proportion of health risk behavior and a higher risk of experiencing violence and accidents as well as premature death (1). Hence, ID are dependent on unrestricted access to healthcare. However, the above-mentioned restrictions of access to healthcare contribute significantly to the poorer overall health status of ID compared to non-disabled people (1). Moreover, ID experience stigma and discrimination in different ways (30). We consider any restriction of access to healthcare as a form of stigmatization. Stigma can be associated with the experience of minority stress, which can lead to depressive and anxiety symptoms (31). Therefore, limiting access to healthcare can in turn itself increase the risk of further health disorders.

### 4.6. Limitations

There are some limitations to our study. First, we included only cases in which any degree of disability officially has been acknowledged. Moreover, we included all cases irrespective of the type or degree of disability the applicant was suffering from. As disability is an extremely variable concept and the criteria for acknowledging a disability status may vary in different states, our analyzed judgments dealt with a variety of health-related phenomena. Second, the judgments included in the analysis do not represent all examples of restricted access to healthcare for ID. The ECtHR only considers cases that run through all domestic instances. Furthermore, it was not possible for us to make an explicit analysis of the illnesses underlying the disabilities in the individual cases. This was due to the fact that (1) often more than one illness was mentioned and (2) it was not possible to identify in individual cases which illnesses led to the granting of disability status. Finally, our categories were overlapping, making a clear assignment of the complex cases to a particular category a difficult task. The assignment of some cases might be disputable.

### 4.7. Conclusion

Our research shows that ID suffer from restricted access to healthcare all over Europe, especially through denial of medical treatment and/or examinations (*n* = 39, 61.82%) or by a denial of adequate support. Indirect ways of a restriction of access to healthcare, such as denial of access to information, denial of insurance or reimbursement, or denial of investigation of complaints should not be overseen. Especially in the context of detention, ID are restricted from receiving the healthcare they require. There is an urgent need to facilitate access to healthcare for ID to prevent further widening of the gap regarding the health status of ID compared to non-disabled persons. As restriction of access to healthcare is in itself a form of stigmatization, it may contribute to ID's poor health.

## Data availability statement

The original contributions presented in the study are included in the article/supplementary material, further inquiries can be directed to the corresponding author.

## Author contributions

TS-E, MO, and FS conceptualized the topic and scope of the research. TS-E and MO performed the data analysis, wrote, and reviewed the original draft. FS supervised the research and reviewed the manuscript. All authors have read and agreed on the submitted version of the manuscript.
